# Evidence of Unique and Generalist Microbes in Distantly Related Sympatric Intertidal Marine Sponges (Porifera: Demospongiae)

**DOI:** 10.1371/journal.pone.0080653

**Published:** 2013-11-12

**Authors:** Anoop Alex, Vitor Silva, Vitor Vasconcelos, Agostinho Antunes

**Affiliations:** 1 CIMAR/CIIMAR, Centro Interdisciplinar de Investigação Marinha e Ambiental, Universidade do Porto, Porto, Portugal; 2 Departamento de Biologia, Faculdade de Ciências, Universidade do Porto, Porto, Portugal; International Atomic Energy Agency, Austria

## Abstract

The diversity and specificity of microbial communities in marine environments is a key aspect of the ecology and evolution of both the eukaryotic hosts and their associated prokaryotes. Marine sponges harbor phylogenetically diverse and complex microbial lineages. Here, we investigated the sponge bacterial community and distribution patterns of microbes in three sympatric intertidal marine demosponges, *Hymeniacidon perlevis*, *Ophlitaspongia papilla* and *Polymastia penicillus*, from the Atlantic coast of Portugal using classical isolation techniques and 16S rRNA gene clone libraries. Microbial composition assessment, with nearly full-length 16S rRNA gene sequences (ca. 1400 bp) from the isolates (n = 31) and partial sequences (ca. 280 bp) from clone libraries (n = 349), revealed diverse bacterial communities and other sponge-associated microbes. The majority of the bacterial isolates were members of the order *Vibrionales* and other symbiotic bacteria like *Pseudovibrio ascidiaceiocola*, *Roseobacter* sp., *Hahellaceae* sp. and *Cobetia* sp. Extended analyses using ecological metrics comprising 142 OTUs supported the clear differentiation of bacterial community profiles among the sponge hosts and their ambient seawater. Phylogenetic analyses were insightful in defining clades representing shared bacterial communities, particularly between *H. perlevis* and the geographically distantly-related *H. heliophila*, but also among other sponges. Furthermore, we also observed three distinct and unique bacterial groups, *Betaproteobactria* (∼81%), *Spirochaetes* (∼7%) and *Chloroflexi* (∼3%), which are strictly maintained in low-microbial-abundance host species *O. papilla* and *P*. *penicillus*. Our study revealed the largely generalist nature of microbial associations among these co-occurring intertidal marine sponges.

## Introduction

Sponges (Phylum Porifera) are an ancient groups of invertebrate metazoans, with a fossil record dating back to 600 million years and with a host of diverse symbiotic microorganisms such as archaea [1], cyanobacteria [2], heterotrophic bacteria [3], algae, fungi [4] and dinoflagellates [5]. Transmission of symbiotic bacteria in sponges is considered to occur through the gametes [6]–[8]. However, a few microbes from the surrounding seawater have been determined to associate with sponges [9], suggesting the horizontal acquisition of symbionts. Thus, a new model of symbiont acquisition that includes both larval-mediated transfer and environmental uptake in the formation of complex sponge microbiota has been proposed [10]. Diverse sponge microbiota may contribute to the host's metabolism with photosynthesis [2], major nitrogen cycle events [11]–[14], sulfate reduction [15] and carbon fixation [16], [17] and in return, the host provides an enriched ecological niche for its microbial partners.

Isolation techniques [18], [19] and several molecular methodologies, such as denaturing gel gradient electrophoresis (DGGE) [20], [21], terminal restriction fragment length polymorphism (T-RFLP) [22], [23] and clone library [24] were used extensively to profile the sponge-derived microbes. To date, 32 major bacterial phyla [25] and several possible novel, sponge-associated bacterial communities, such as “*Candidatus* Synechococcus spongiarum” [26], candidate phylum “*Poribacteria*” [27] and NW001-like *Alphaproteobacteria* [8], have been identified. In addition, with the advancement of sequencing techniques (i.e., next generation sequencing) [9], researchers have succeeded in exploring diverse microbial consortium among a wide range of sponge hosts [28]. The search for sponge-associated bacteria has been intensified given the pharmaceutical potential of novel bioactive compounds [29]. However, little is known about the driving evolutionary force behind the specificity of microbial symbionts.

The ecological assessment of sponge-microbe specificity and the diversity of sponge-associated bacterial communities has recently attained more attention to better understand the global marine biodiversity [30]. Previous studies have shown that sponge-bacterial symbioses range from associating with a few or single host species (specialism) [23], [31] to many hosts (generalism) [32] in time and space. Difference in microbial abundance separated the sponges into low-microbial-abundance (LMA) and high-microbial-abundance (HMA) groups [33]. LMA sponges host less-diverse and more-specialist microbial communities [23], [34], whereas HMA sponges harbour diverse and distinct bacterial communities. Additional data on bacterial diversity is needed to better describe sponge-microbe associations and in this study we examined the bacterial diversity in three co-occurring intertidal sponges from the Atlantic Ocean in Portugal: *Hymeniacidon perlevis* Montagu, 1818 (Halichondrida: Halichondriidae), *Ophlitaspongia papilla* Bowerbank, 1866 (Poeciosclerida: Microcionidae) and *Polymastia penicillus* Montagu, 1818 (Hadromerida: Polymastiidae).

The distribution of *H*. *perlevis* (crumb-of-bread sponge) ranges from the Atlantic to the Mediterranean coast and in the Yellow Sea, inhabiting hard surfaces that are buried into the sediment, and with their oscules projecting outwards [35]. A high microbial diversity of *Actinobacteria* was reported in *H. perlevis* distributed in the Chinese Yellow Sea by culture-dependent [36] and culture-independent techniques [37]. *O. papilla* is a deep orange-red colored sublittoral sponge previously reported from the British Isles, France and Spain [38], but the species can also be found in Portugal. It has been studied because of its cytotoxic activities [39], but no evidence has been found for the existence of bacterial communities. *P. penicillus* commonly inhabit cervices, with their main body buried in sediments and papillae projecting outward. Its distribution ranges from the British Isles and the Atlantic coasts of Europe and North America and it has recently been shown to produce pharmacologically active secondary metabolites [40].

The marine sponges studied here represent three distantly related orders of the Demospongiae class, living sympatrically in the clear intertidal ecological zone with frequent exposure to air during low tide in contrast with other constantly submerged species. Despite the existence of some sponge-associated microbial studies across different geographic regions (Mediterranean [10], [32], [41], Australia [9], [30], Caribbean [42], [43] and Southwestern Atlantic [1], [24]), to our knowledge no similar characterizations have been done in the Atlantic Ocean (European Atlantic coast). Here, we used bacterial isolation and culture-independent techniques (16S rRNA gene clone library) to characterize the sponge microbial profiles, distinct microbial community structure and the distribution of symbiotic microbes in three sympatric intertidal marine sponges from a single geographical location (Praia da Memória, Portugal).

## Results

### Phylogenetic relationships of the bacterial isolates from sponges

**Figure 1:**
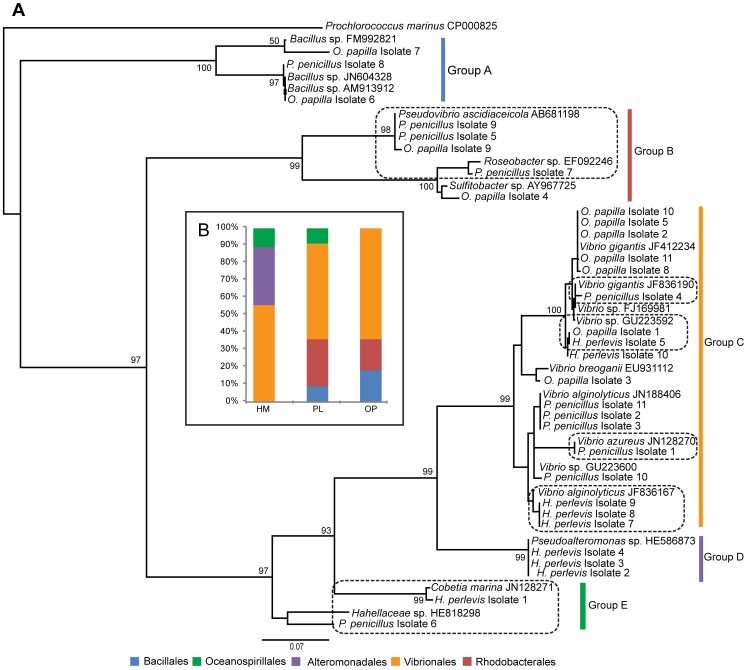
Phylogeny and diversity of bacterial isolates from marine sponges *H. perlevis* (HM), *P. penicillus* (PL) and *O. papilla* (OP). (**A**) Maximum-likelihood phylogenetic tree of nearly full length 16S rRNA gene sequences (ca. 1400 bp) using *Prochlorococcus marinus* as an outgroup. Sponge-derived bacterial isolates obtained from this study are highlighted in bold. The closest relatives retrieved through the BLAST search (Table S1) with their GenBank accession numbers are represented. Dashed box delimit the sponge-associated bacterial groups (i.e. the grouping of bacteria retrieved from this study with the previously reported microbes from other sponges). Bootstrap node support values >50% are represented. (**B**) Stacked histogram showing the relative abundance of 16S rRNA diversity recovered from the sponge sources.

Culture-dependent techniques allowed the isolation of heterotrophic bacteria from the marine sponges *H. perlevis* (n = 9), *O. papilla* (n = 11) and *P. penicillus* (n = 11). BLAST searches with nearly full-length 16S rRNA sequences (ca. 1400 bp) from the isolates identified both Gram-positive bacteria *Firmicutes* (10%) and Gram-negative group *Proteobacteria*, predominantly *Alphaproteobacteria* (16%) and *Gammaproteobacteria* (74%) (Figure 1, Table S1). Phylogenetic analyses further confirmed the presence of a group of bacterial isolates (45%) overlapping with previously-reported sponge and other marine invertebrate-associated bacteria. *P. penicillus* and *O*. *papilla* bacterial isolates had strong affiliation with the ascidian symbiont *Pseudovibrio ascidiaceicola* (n = 3) and the sponge-associated *Roseobacter* sp. (n = 1). Most of the cultured bacterial assemblages clustered within the order *Vibrionales*. Isolates affiliated to *Oceanospirillales* (previously reported in marine sponges) were also retrieved from *H. perlevis* and *P. penicillus*.

### 16S rRNA clone library and OTU abundance

**Figure 2:**
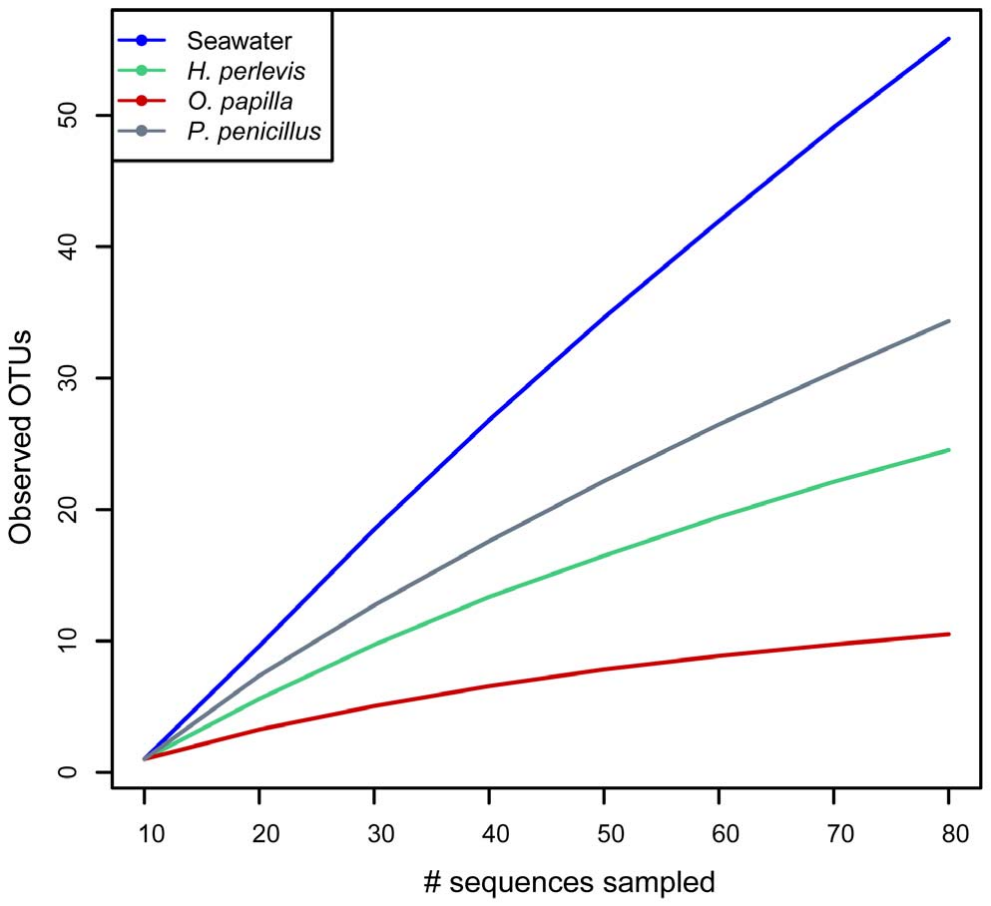
Rarefaction curve for 16S rRNA gene sequences derived from seawater, *H*. *perlevis*, *O*. *papilla* and *P*. *penicillus*. OTUs are retrieved at a distance of 0.01(99% similarity). Each line represents the respective sponge species and surrounding seawater used.

A final data set consisting of 349 partial 16S rRNA sequences from the sponge species *H*. *perlevis* (n = 86), *O*. *papilla* (n = 91) and *P*. *penicillus* (n = 77) and surrounding seawater (n = 95), were assigned to 142 Operational taxonomic units (OTUs) at 0.01distance. Clustering bacterial sequences from each source at lower identity thresholds resulted in a reduced number of observed OTUs (Figure S1). Rarefaction curves (Figure 2) and *Chao1* (Table S2) suggested higher bacterial richness in ambient seawater (300) followed by the host sponges *P*. *penicillus* (181), *H*. *perlevis* (45) and *O*. *papilla* (15). Good's coverage estimator (Table S2) indicated the completeness of sampling for *O. papilla* (0.81), less clone sampling for *H. perlevis* (0.60), *P. penicillus* (0.42) and seawater (0.30). Seawater OTUs remained non-saturated suggesting much more bacterial species diversity remains to be discovered.

### Uncultured sponge microbiota composition

16S rRNA ribotype diversity retrieved from the marine sponges *H*. *perlevis*, *O*. *papilla* and *P*. *penicillus*, revealed bacterial community representatives of 10 phyla (Table 1). *Alphaproteobacteria* and *Bacteroidetes* were recovered from all three host sponges and seawater sources. *H. perlevis* was dominated by *Gammaproteobacteri*a (67.4%) and *Alphaproteobacteria* (24.5%). *O. papilla* was dominated exclusively by *Betaproteobacteria* (81.3%) as a major and unique group of bacteria (Figure S2, Table 1). The members of the phylum *Alphaproteobacteria* were predominant in *P. penicillus* (80.5%), but rare in *O. papilla* (5.49%). Among the host sponges and seawater sampled, *Betaproteobacteria* and *Spirocheates* were only retrieved from *O. papilla*. Another member of the phylum *Chloroflexi* (2.59%) was found in low abundance associated with the sponge *P. penicillus*, but absent in the other sources.

**Table 1:** Bacterial community assemblage retrieved from seawater and sponges, shown as percentage.

	Seawater (n = 95)	*H. perlevis* (n = 86)	*O. papilla* (n = 91)	*P. penicillus* (n = 77)
Actinobacteria	3.15	2.32	-	-
Alphaproteobacteria	**36.8**	**24.4**	5.49	**80.5**
Bacteroidetes	**21.08**	3.48	3.29	5.19
Cyanobacteria	3.15	-	2.19	-
Clostridia	1.05	-	-	-
Deltaproteobacteria	3.15	1.16	-	3.89
Epsilonproteobacteria	2.10	-	-	-
Fusobacteria	7.36	-	-	-
Gammaproteobacteria	**15.78**	**67.4**	-	5.19
Archaea	2.1	-	-	-
Planctomycetia	2.1	1.16	-	2.59
Unclassified bacteria	1.05	-	-	-
Verrucomicrobiae	1.05	-	-	-
Betaproteobacteria	-	-	81.3*	-
Spirochaetes	-	-	7.69*	-
Chloroflexi	-	-	-	2.59*

Total number of clones from each source is represented in parenthesis. Values in bold represent the most dominant bacterial group found in association with source. Values with asterisk show the unique bacterial lineage from sponges.

### Bacterial diversity and community structure

**Figure 3:**
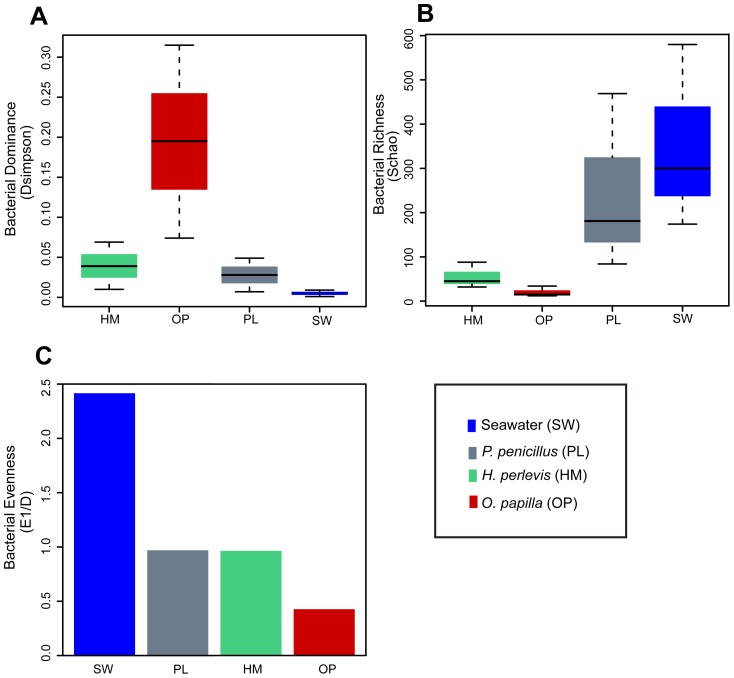
Ecological diversity metrics of bacterial communities from *H. perlevis*, *O. papilla*, *P*. *penicillus* and seawater. (A) Bacterial dominance (B) Bacterial richness and (C) Bacterial evenness.

**Figure 4:**
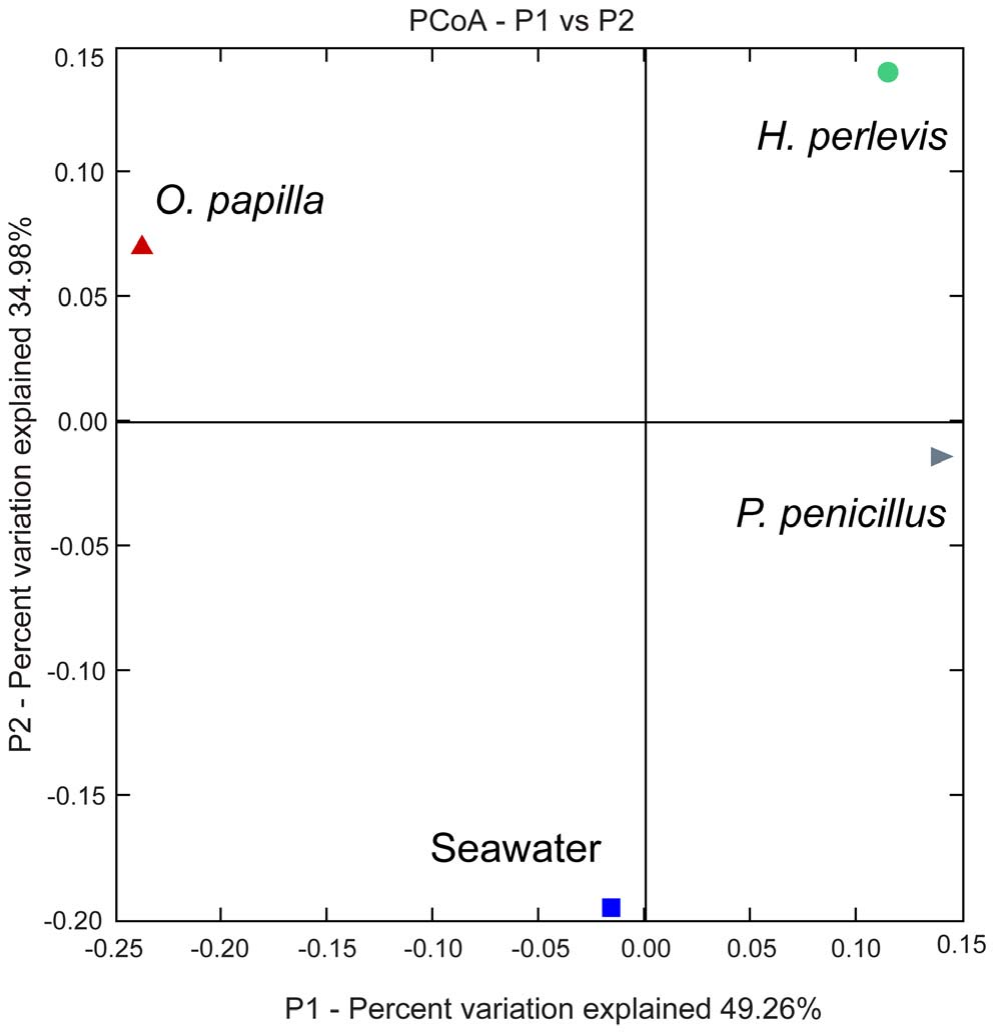
Principal Coordinate Analysis (PCoA) plot based on weighted unifrac distances. 16s rRNA sequences are binned according to sample source using a category mapping file. The percentage variation explained with first two principal components (P1 and P2).

**Figure 5:**
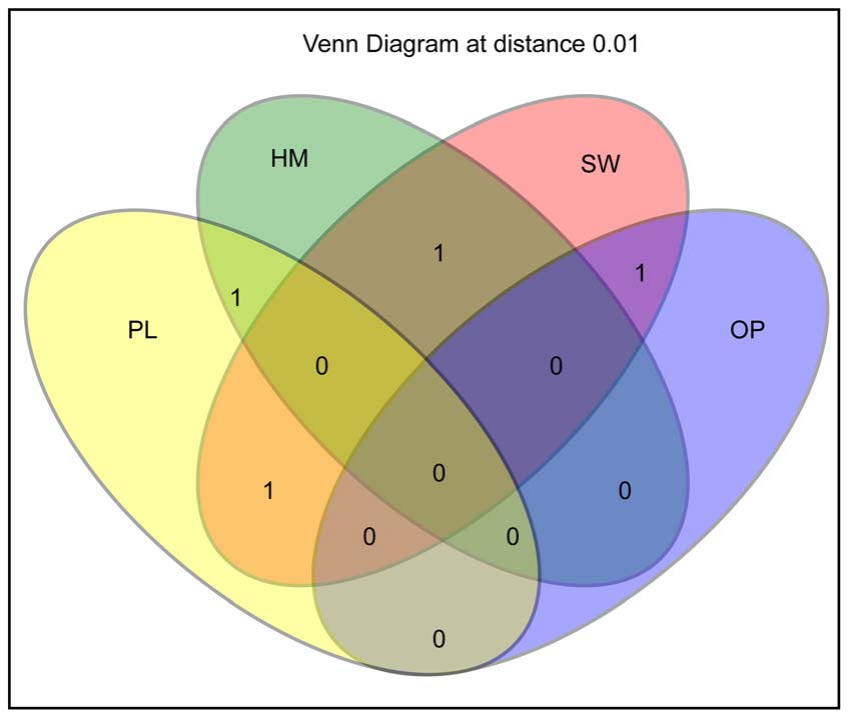
Venn diagram representing the distribution of shared OTUs among sponge hosts *H*. *perlevis* (HM), *O*. *papilla* (OP), *P*. *penicillus* (PL) and seawater (SW). The numbers in the diagram represents unique and shared OTUs.

Quantitative ecological indices revealed significant differences in bacterial communities among the various sources. Seawater-bacterial communities exhibited higher observed (*S_obs_*) and expected (*S_chao1_*) richness, lower dominance (*D_simpson_*) and higher evenness (*E_1/D_*) indices indicating a clear bacterial differentiation among the sponge-associated bacterial communities (Figure 3, Table S2). Similar evenness values were observed among the bacterial communities in *P*. *penicillus* and *H*. *perlevis*. In addition, these two sponges showed an overlapping confidence interval indicating similar microbial diversity. The homogeneity of the molecular variance test confirmed a significant higher genetic diversity among seawater-associated bacterial communities (_HOMOVA_, *P*<0.005) (Table S3) relative to the sponge-associated bacterial communities. Pairwise comparison showed significant genetic differentiation (_AMOVA_, *P*<0.001) and distinct phylogenetic lineages (P- test, *P*<0.001) among bacterial communities from different sources (Table S3). LIBSHUFF analysis clearly indicated that the bacterial community structure was different (LIBSHUFF, *P*<0.0001) between the host sponges (Table S3). Principal Coordinate Analysis (PCoA) revealed the distinctiveness of bacterial communities from the sponge hosts and seawater (Figure 4). Seawater clones shared only one OTU with the host sponges (Figure 5). By contrast, *H. perlevis* and *P. penicillus* shared one OTU and none between other sponges.

### Phylogenetic analysis of the uncultured sponge microbiota

**Figure 6:**
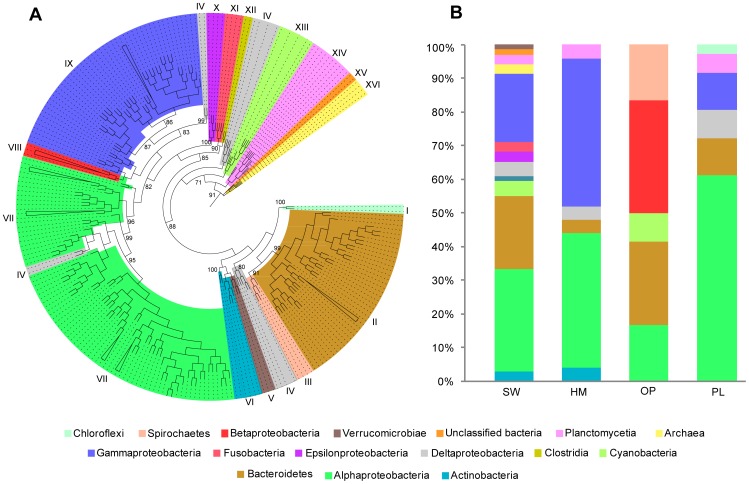
Phylogeny and diversity of 16S rRNA gene clone libraries from *H. perlevis*, *O. papilla*, *P*. *penicillus* and seawater. (A) Maximum-likelihood circular phylogenetic tree constructed using partial 16S rRNA gene sequences bacterial OTUs. (B) Clone library diversity inferred from each source.

**Figure 7:**
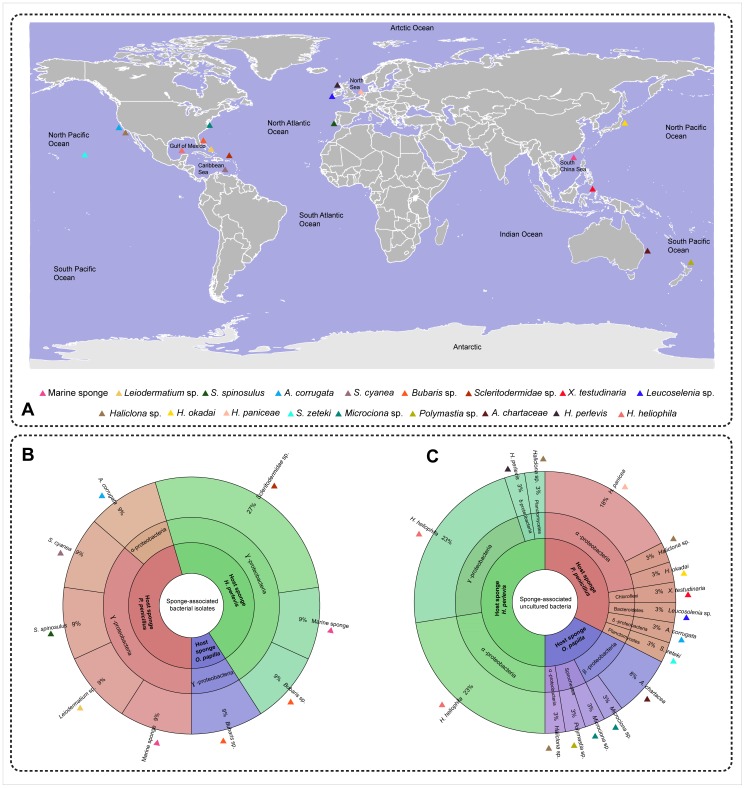
Sponge-associated bacteria among the host sponges *H. perlevis*, *P. penicillus* and *O*. *papilla* and its similarity with globally distributed related and unrelated host sponges. (A) Global map depicting the location of host sponges harboring bacterial assemblage similar to the retrieved microbes from the present study. Colored triangle on the map shows the sponge species from other studies. (B–C) Sponge associated bacteria from different hosts determined by isolation and uncultured techniques, represented as a pie chart [73] showing its abundance and affiliation with microbes found in association with globally distributed host sponges.

Phylogenetic analysis with OTUs (n = 142) from seawater and sponges confirmed the existence of distinct bacterial groups (Figure 6). The major groups II, VII and IX comprised sequences representative of *Bacteroidetes* (16.1%), *Alphaproteobacteria* (38.7%) and *Gammaproteobacteria* (20.4%), but *Gammaproteobacteria* was absent in the sponge *O. papilla.* Clones from *P. penicillus* (22%) in group VII were affiliated with symbionts from the sponge *Halichondria panicea* from the North Sea (Figure 7) and in a few cases with symbionts from the sponge *Haliclona* sp. and the brittle star (*Ophiactis balli*). Notably, both the *H. perlevis* clones in group VII (36%) and clones in group IX (37%) were affiliated with the symbionts reported from the geographically separated congeneric sponge *H. heliophila* from the Gulf of Mexico.

*Bacteroidetes* in group II, represented by seawater clones (65%), were related to the sediment coastal water and biofilm bacteria. However, *P. penicillus* (clone 58) shared similarity with the bacterial strain (JX050191, group II) from the marine sponge *Leucosolenia* sp. (class Calcarea) (Figure S3). Other sponge-derived bacterial clones affiliated to the major phyla are represented in group I (*Chloroflexi*), group III (*Spirochaetes*), group IV (*Deltaproteobacteria*), group VIII (*Betaproteobacteria*) and group XIV (*Planctomycetia*). Our phylogenetic analysis recurrently revealed an overlap of the microbes detected in this study with sponge-associated bacteria previously reported elsewhere. It is noteworthy that none of the 16S rRNA clone library sequences retrieved from the studied sponges showed similarity with the cultured bacterial isolates.

## Discussion

### Bacterial isolates from the sponges

In this study we determined the diversity of the cultivable bacteria associated with the sponges using classical culturing/isolation techniques. The cultured heterotrophic bacterial isolates (>35%) from the host sponges *H. perlevis*, *O*. *papilla* and *P*. *penicillus* showed high similarity with previously described sponge-associated bacteria from different geographical locations (Figure 7). The isolation of *P*. *ascidiaceicola* from the sympatric sponges *O. papilla* and *P. penicillus* (Table S1), with high sequence similarity (>99%) with the isolate from the sea squirts *Polycitor proliferus* and *Botryllidae* sp. [44], suggests a symbiotic lifestyle with a wide range of hosts. A wide diversity of *Pseudovibrio* sp. have been isolated from the sponges in Ireland [45], Mediterranean Sea [46] and Brazil [47] with promising antimicrobial activity. The absence of sea squirts and retrieval of *P*. *ascidiaceicola* isolates from two sponges studied here suggests a symbiotic association with the intertidal marine sponges. The specificity of *P*. *ascidiaceicola* in the intertidal sponges should, however, be better assessed by sampling simultaneously the ascidians and sponges coexisting in the same location and characterizing the bacterial isolates. Genome sequencing of two strains of the genus *Pseudovibrio* revealed the gene clusters that likely to be involved in host-symbiont interactions [48].

Approximately 58% of the cultured bacterial isolates from the three host sponges studied here grouped into *Vibrionales* (Figure 1), with high sequence similarity to other sponge-derived (SD) *Vibrio* isolates. The high affinity of vibrios from the intertidal (shallow) marine sponges with benthic SD vibrios from *Leiodermatium* sp. and *Scleritoderma cyanea*, collected at a depth of 630 m and 364 m from globally distinct locations [49], demonstrates the ubiquity of Vibrios among the sponge hosts. The higher incidence of *Vibrio* sp. has been also reported in other marine sponges [50], as well as the occurrence of *V*. *alginolyticu*s as probionts [51]. Sponge-associated *Vibrio* sp. might benefit the host through nitrogen-fixation [52], but the precise role is still unclear. *Pseudoalteromonas* sp. was isolated from *H. perlevis*, which has been shown previously to degrade polycyclic aromatic hydrocarbon (PAHs) [53]. The exact role of *Pseudoalteromonas* sp. is unclear, but it may protect the sponge host from toxic environments. We also isolated other sponge-associated bacteria, *Hahellaceae* sp. and *Cobetia marina* (*Oceanospirillales*), that have been associated with the Atlantic-Mediterranean sponge *Sarcotragus spinosulus* and marine sponges from the South China Sea (Figure 7). Here, we report the bacterial isolates from *H. perlevis*, *P. penicillus* and *O. papilla* and their similarity to sponge-associated bacteria from unrelated hosts and remote locations.

### 16S rRNA clone library diversity and specificity

The bacterial community profile determined by 16S rRNA clone libraries from three sympatric intertidal marine sponges, *H. perlevis*, *O. papilla* and *P. penicillus*, was noticeably distinct from the surrounding seawater bacterial assemblage (Figure 4). Ecological metrics, such as higher dominance, lower richness and lower evenness of the sponges (Figure 3) further supported a clear distinction between the microbes from the sponge hosts and the ambient seawater. Rarefaction analysis and coverage estimation showed less sampling depth of bacterial species from *H. perlevis* and *P. penicillus*, relative to *O. papilla* (Figure 2, Table S2). Considering the complex nature of sponge-associated bacterial assemblages, deep sequencing would avoid an underestimation of the bacterial diversity. Intensive sampling would likely reveal more bacterial species from the respective sponge hosts studied. Quantitative assessment of the sponge-associated bacterial diversity revealed by our molecular approach suggests that the sponges assessed here are low-microbial-abundance (LMA) species. However, a combination of electron microscopy to determine the bacterial morphotypes and molecular techniques could better answer the microbial abundance in the sponge hosts [24].

In addition, absence or minimal sharing of OTUs (Figure 5) among the sponges collected from the same sampling location (10 m apart between each collection site) suggests the existence of unique microbial communities. Around 50*%* of the sponge-associated bacterial 16S rRNA retrieved from our study showed similarity with other sponge-associated bacteria reported elsewhere, affirming the widespread nature of the sponge microbial consortium (Figure 7). The existence of certain observed bacterial lineages in sympatric sponges indicates the possibility of microbial procurement through the vertical transmission of symbionts via larvae or from the water column (horizontal uptake). Some of the bacteria found in sponges had strong affiliation with the sediment bacteria, which might be either filtered food-particles or occasional resident bacteria. The detection of many sponge-associated bacteria in non-sponge habitats suggests the survival of bacteria outside the sponge body and uptake from the surrounding through filter feeding [54]. Next generation sequencing techniques and more sampling data could provide valuable information to better understand the nature of such associations.

Among the three sympatric intertidal marine sponges, the majority (76%) of microbial communities retrieved from *H*. *perlevis* showed similarity with the 16S rRNA sequences associated with the sister species *H*. *heliophila* (Figure 7, Figure S3-group VII and group IX) sampled from the Gulf of Mexico [23]. Interestingly, none matched with the *H*. *heliophila* associated bacteria sampled from southwestern Atlantic of Brazil [24]. Absence of similarity with bacterial clones from the southwestern Atlantic congeneric species might be due to (i) insufficient 16S rRNA sequence sampling or (ii) the influence of different environmental factors.

Furthermore, we observed a clear global distribution pattern (homogenous pattern) of microbial assemblage retrieved from the three sympatric sponges investigated in this study (Figure 7). For example, *Planctomycetacia* (group XIV) from *H. perlevis* and *P. penicillus* showed high similarity with the bacterial sequences associated with the marine sponges *Haliclona* sp. and *Suberites zeteki* (Figure S3). *H*. *perlevis* and *P. penicillus* associated bacteria formed a sequence cluster with the bacterial sequences derived from the unrelated sponge *Axinella corrugata*, suggesting a generalist microbial distribution among sponges. A bacterium affiliated with the phylum *Chloroflexi* [6], a common lineage of sponge-associated bacteria, was retrieved only from the clone library of *P*. *penicillus* (Figure 6, Table 1), and in low abundance (2.59%). Interestingly, the *P*. *penicillus* bacterial community included *Maribacter* sp. W1510, which has been previously isolated from the marine sponge *Leucosolenia* sp. (class *Calcarea*). Further investigation is needed to confirm the microbial community similarity among sponges from different classes.

Consistent with the previous findings, we observed a trend of unique and generalist bacterial associations in the encrusting sponge *O*. *papilla*. Clone library analysis revealed that *Betaproteobacteria* (81.3%) was an abundant and unique microbial lineage and that the less abundant *Spirochaetes* (7.69%) was associated with *O*. *papilla* (Figure 6), forming two distinct clusters (Figure S3; group VIII and group III) with other sponge-associated bacteria in a pattern that is suggestive of a generalist. Further studies, including more extensive sampling, are required to confirm if the uniqueness of the bacterial assemblages are observed in other sampling periods. Under-representation of the cyanobacterial sequences from the sponges might be due to either the reduced number of clones tested or the absence of photosynthetic bacteria during the sampling period. We also observed no archaea bacteria despite the use of archaea and bacterial specific primers. This is perhaps due to either primer inefficiency or inadequate clone sampling.

### Generalist bacterial diversity – A common trend among sponge microbiota

The culture-dependent and culture-independent methodologies retrieved a bacterial consortium from the intertidal marine sponges *H. perlevis*, *P. penicillus* and *O. papilla* with a trend of both ‘unique’ (low tendency) and ‘generalist’ (high tendency) associations. A higher tendency towards generalist association of the bacterial symbionts was evident among the sympatric sponges studied. Various ecological parameters, including physical factors like season, chemical variables (pH of surrounding water) and nutrient needs, might play an important role in determining the sponge-microbial structure [30]. Variation in the photosynthetic bacterial communities has been reported among the studied sponges *H. perlevis* sampled during different seasons [55]. The occasionally submerged sponges studied, which are exposed to constant water movement and occasional sun light, may impose selective pressures that shape the microbial consortium within the sponges. Extensive sampling from different geographical locations is needed to investigate the spatial-temporal structuring of the microbiota.

In conclusion, we characterized for the first time the bacterial communities in three sympatric intertidal marine sponges *H. perlevis*, *O. papilla* and *P*. *penicillus* from the Atlantic coast, confirming unique and diverse microbes from 10 different bacterial phyla. The low-microbial-abundance sponge species *O. papilla* and *P*. *penicillus* harbored unique microbes compared with other co-occurring sponge. The majority of the bacterial 16S rRNA sequences in this study were similar to other related and unrelated sponge-associated/non-sponge associated bacteria, irrespective of depth and geography, further evidence of the complex nature of the associations. These new results add insights on the global diversity of sponge-associated bacteria. However the full nature of the observed associations will require further detailed investigations.

## Materials and Methods

### Ethics statement

The study did not involve any endangered or protected species. No specific scientific research permits were required for invertebrate sample collection from the intertidal rocky beach. Moreover, sponge sample collection did not require the sacrifice of the animal and only 1 cm^3^ of the inner region of sponge tissue was used for the study, which can be readily regenerated by the animal and have no environmental effect.

### Sample collection and processing for culturable bacteria

Marine sponge species *Hymeniacidon perlevis* (n = 2), *Ophlitaspongia papilla* (n = 3) and *Polymastia penicillus* (n = 2) were collected from the intertidal rocky shore, Praia da Memória in Portugal (41.2308206N 8.7216926W). Sponge specimens and surrounding seawater were collected and transported in an insulated cooler to lab for immediate procedure. Samples were rinsed with sterile seawater by gentle shaking 4 to 5 times to remove loosely attached superficial bacteria. Approximately 1 cm^3^ inner region of the sponge tissue was dissected and grinded using a sterile mortar and pestle in 10 ml autoclaved seawater. The homogenate was serially diluted to 10^−5^ and plated on Difco™ Marine Agar 2216 medium. Amphotericin B (1 ml/100 ml) was added to medium to prevent the fungal growth. Plates were incubated at 28°C for up to 1 week in dark. Colonies were re-streaked twice onto the medium to obtain the pure culture.

### Identification of the cultured bacteria by 16S rRNA gene

Distinct bacterial colonies were screened based on morphology and color for further analyses. For DNA extraction, pure bacterial colonies were inoculated in 2 ml tubes containing Difco™ Marine broth 2216 medium. Culture tubes were incubated at 28°C with constant shaking for 3 days in dark for maximum bacterial cell harvest, followed by DNA extraction using PureLink™ Genomic DNA kit (Invitrogen) according to manufactures instruction.

16S rRNA genes were amplified with universal bacterial primers 8F (5′-AGAGTTTGATCCTGGCTCAG -3′) and 1492R (l) (5′-GGTTACCTTGTTACGACTT-3′) [56]. PCR reactions were performed in 50 µl volume, consisting 5 µl of 10× PCR buffer (Invitrogen), 5 µl of 2.5 mM DNTPs, (NZYTech), 2.5 µl of 2.5 mM MgCl_2_ (Invitrogen), 2.5 µl of 10 µm each primer, 0.5 µl of 5 U/µl *Taq* DNA polymerase (Invitrogen) and 2.5 µl of 30 ng genomic DNA. PCR cycling profiles: initial denaturation for 5 minutes at 94°C; 30 cycles of 94°C for 1 min, 55°C for 1 min, 72°C for 2 min; and a final extension for 10 minutes at 72°C. PCR products were purified with PureLink™ Quick Gel Extraction and PCR Purification Combo Kit (Invitrogen) and sequenced by the Macrogen Europe using an ABI 3730XL DNA Analyzer (Applied Biosystems). Sequences were checked for chimeras using Mallard [57] and were deposited in GenBank (accession numbers KF155235–KF155265).

### DNA extraction from the sponge and surrounding seawater

Sponge species *H. perlevis* (n = 2, abbreviated as HM), *P. penicillus* (n = 2, abbreviated as PL) and *O. papilla* (n = 3, abbreviated as OP) were processed (described in previous section) for total genomic DNA. About 1 cm^3^ sponge tissues was digested according to the protocol specified for the gram positive and gram negative bacteria with the PureLink™ Genomic DNA kit (Invitrogen). Surrounding seawater (n = 1, abbreviated as SW) was filtered through sterile 0.45 µm pore size filter. Particles attached to the filter paper were scratched out following DNA extraction. Molecular identification of the sponge species were performed by using gene marker cytochrome oxidase I (COI). Sponge barcoding primers [58] were used to amplify a partial region of COI using previously described PCR conditions [55]. Sequences were deposited in GenBank (accession numbers KF225481–KF225487).

### PCR and 16S rRNA gene library construction

Partial 16S rRNA gene was amplified for clone library preparation with the universal forward primer U789F (5′-TAGATACCCSSGTAGTCC-3′) and reverse primer U1068R (5′-CTGACGR CRGCCATGC-3′) targeting the hypervariable region V6 of both bacteria and archaea [59]. Triplicate PCR reactions were performed for each sponge specimens in 50 µl volume, consisting of similar PCR reaction mixture used for culturable bacteria except an increase in primer concentration (5 µl of 10 µm each primer), with previously reported cycling conditions [28]. PCR products were gel purified with PureLink™ Quick Gel Extraction and PCR Purification Combo Kit (Invitrogen).

Purified respective 16S rRNA amplicons were pooled and used as DNA insert for clone library construction using pTOP TA V2 vectors in Macrogen Europe. Positive clones were screened, and clones with ∼300 bp insert were sequenced bidirectional using vector primers.

Raw sequence data from 384 clones were searched for vector contamination using VecScreen (http://www.ncbi.nlm.nih.gov/VecScreen/VecScreen.html). The sequences were further validated and trimmed to about 300 bp with a sequence preprocessing pipeline, SeqTrim [60], and if necessary manually inspected and edited in BioEdit Sequence Editor [61]. The processed sequences were checked for chimeras and other artifacts using Mallard [57]. Chimera removed dataset (n = 349) were used for further analyses and deposited in GenBank (accession numbers KF171537–KF171885).

### Operational taxonomic unit (OTU) assignment for clone library

Clone library sequences were aligned using Nearest Alignment Space Termination (NAST) algorithm [62] implemented in Greengenes 16S rRNA gene database [63]. Sequences were assigned and clustered to OTUs at different cutoff values using furthest neighbor method implemented in mothur software package [64]. OTUs defined at a cutoff value of 0.01 (99% similarity) were included in subsequent biodiversity and community structure analyses.

### Bacterial biodiversity and community structure analyses of clone library

Biodiversity indices and the genetic structure for recovered bacterial community from host sponges and seawater (SW) were estimated with various calculators implemented in mothur package [64]. Bacterial richness calculation included observed species richness (*S_obs_*) and expected richness with Chao1 estimator (*S_chao1_*). Simpson's diversity indices, *D_simpson_* and E_1/D_ were used to estimate the bacterial dominance and evenness. Good's coverage estimator was used to determine sampling coverage. Genetic differentiation among and within sponge-associated bacterial community and surrounding water was compared using AMOVA [65] and HOMOVA [66]. Similarities among the symbiont community structure were evaluated with phylogenetic lineage sorting test (P-test; [67]). Pairwise comparison of bacterial communities among sponge hosts and seawater was estimated using LIBSHUFF [68]. Two dimensional Principal Coordinate Analysis (PCoA) was performed to visually compare the community dissimilarity among the source using weighted Unifrac algorithm with normalization steps [69].

### Phylogenetic analysis of cultured bacteria and clone libraries

Cultured bacterial 16S rRNA sequences (n = 31) and OTUs from seawater and respective sponge hosts (n = 142) defined at 99% similarity, and its nearest relative obtained from GenBank database using BLAST search in September 2012 were aligned to greengenes reference database [63]. The taxonomic affiliation is further verified with RDP database [70] to minimize the taxonomic misinterpretation. Maximum-likelihood phylogenetic trees were constructed in PhyML [71] with Nearest-Neighbor- Interchange (NNI) heuristic search method. The best fit evolutionary models, GTR+I+G for isolates and TIM+G+I for clone libraries were adopted under Akaike Information Criterion with correction (AICc) implemented in MrAIC ver. 1.4.4 [72].

## Supporting Information

Figure S1 **OTU diversity of bacterial communities retrieved from three sponge hosts** ***H. perlevis*** **,** ***O*** **.** ***papilla*** **and** ***P*** **.** ***penicillus*** **and surrounding seawater.** OTUs are defined at different similarity threshold.
(DOCX)

Figure S2 **Relative abundance of clones distributed in seawater and sponge hosts** ***H. perlevis*** **,** ***O*** **.** ***papilla*** **and** ***P*** **.** ***penicillus*** **.** A total number of clones retrieved (n = 349) from all source are classified at Domain/Phylum/Class level.
(DOCX)

Figure S3 **Maximum-likelihood phylogenetic tree constructed with partial 16S rRNA gene derived from sponge-associated bacteria.** The closest relative retrieved from database was used for tree construction. Different bacterial groups are shown in colored rectangular box. Clone sequences derived from this study are represented in bold followed by the clone number. The clades are condensed (triangles) and bootstrap support values (%) are indicated.
(PDF)

Table S1 **Identified bacterial isolates from three sponges,** ***H. perlevis*** **,** ***O. papilla*** **and** ***P. penicillus*** **.** The closest relative for each isolate was retrieved showing highest percent of similarity through BLAST search.
(DOCX)

Table S2 **Species richness, dominance, evenness and sampling coverage indices for bacterial communities from seawater,** ***P*** **.** ***penicillus*** **,** ***H*** **.** ***perlevis*** **and** ***O*** **.** ***papilla*** **(upper and lower confidence interval in parentheses).**
(DOCX)

Table S3 **Bacterial genetic diversity and community structure of sponges -** ***O*** **.** ***papilla*** **(OP),** ***H*** **.** ***perlevis*** **(HM),** ***P*** **.** ***penicillus*** **(PL) and seawater (SW).**
(DOCX)
