# Supplementary material for: Evidence of Unique and Generalist Microbes in Distantly Related Sympatric Intertidal Marine Sponges (Porifera: Demospongiae)
Source: PLoS One. 2013 Nov 12;8(11):e80653. doi: 10.1371/journal.pone.0080653 (PMC3827218; doi:10.1371/journal.pone.0080653)
Supplement: Figure S1 — OTU diversity of bacterial communities retrieved from three sponge hosts H. perlevis , O . papilla and P . penicillus and surrounding seawater. OTUs are defined at different similarity threshold. (DOCX) [file pone.0080653.s001.docx]

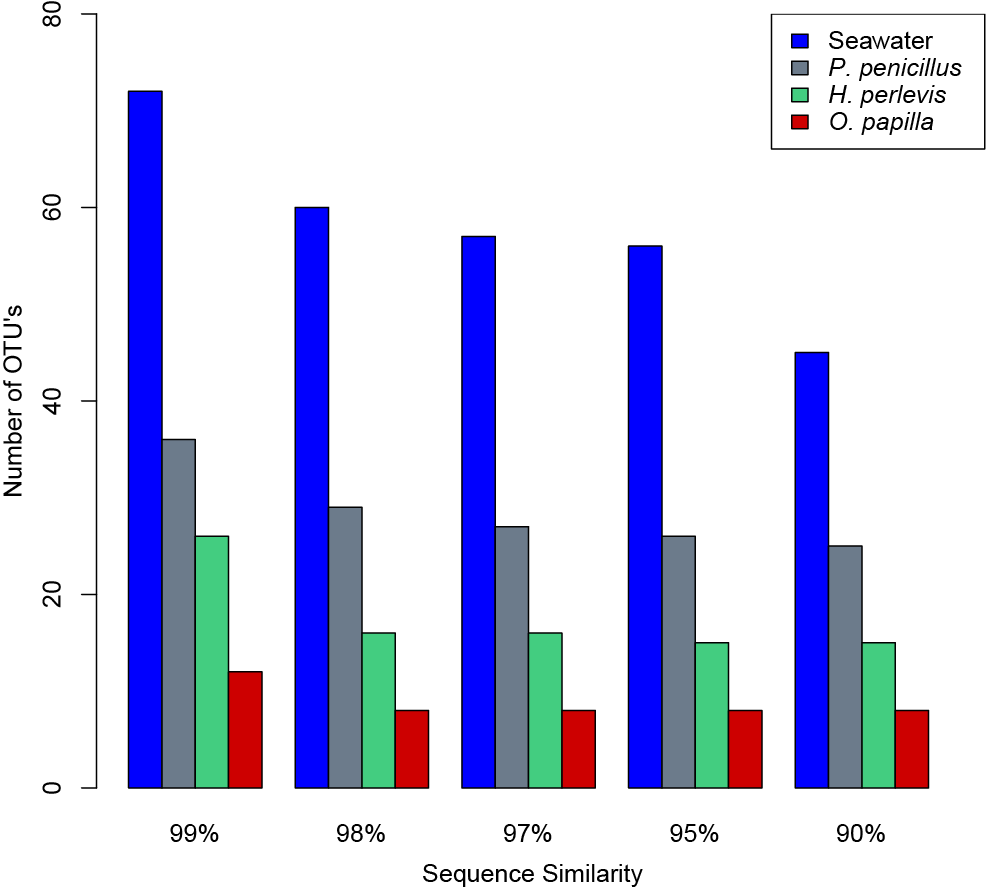


**Figure S1. OTU diversity of bacterial communities retrieved from three sponge hosts *H. perlevis*, *O*. *papilla* and *P*. *penicillus* and surrounding seawater**. OTUs are defined at different similarity threshold.
