# Supplementary material for: Evidence of Unique and Generalist Microbes in Distantly Related Sympatric Intertidal Marine Sponges (Porifera: Demospongiae)
Source: PLoS One. 2013 Nov 12;8(11):e80653. doi: 10.1371/journal.pone.0080653 (PMC3827218; doi:10.1371/journal.pone.0080653)
Supplement: Figure S2 — Relative abundance of clones distributed in seawater and sponge hosts H. perlevis , O . papilla and P . penicillus . A total number of clones retrieved (n = 349) from all source are classified at Domain/Phylum/Class level. (DOCX) [file pone.0080653.s002.docx]

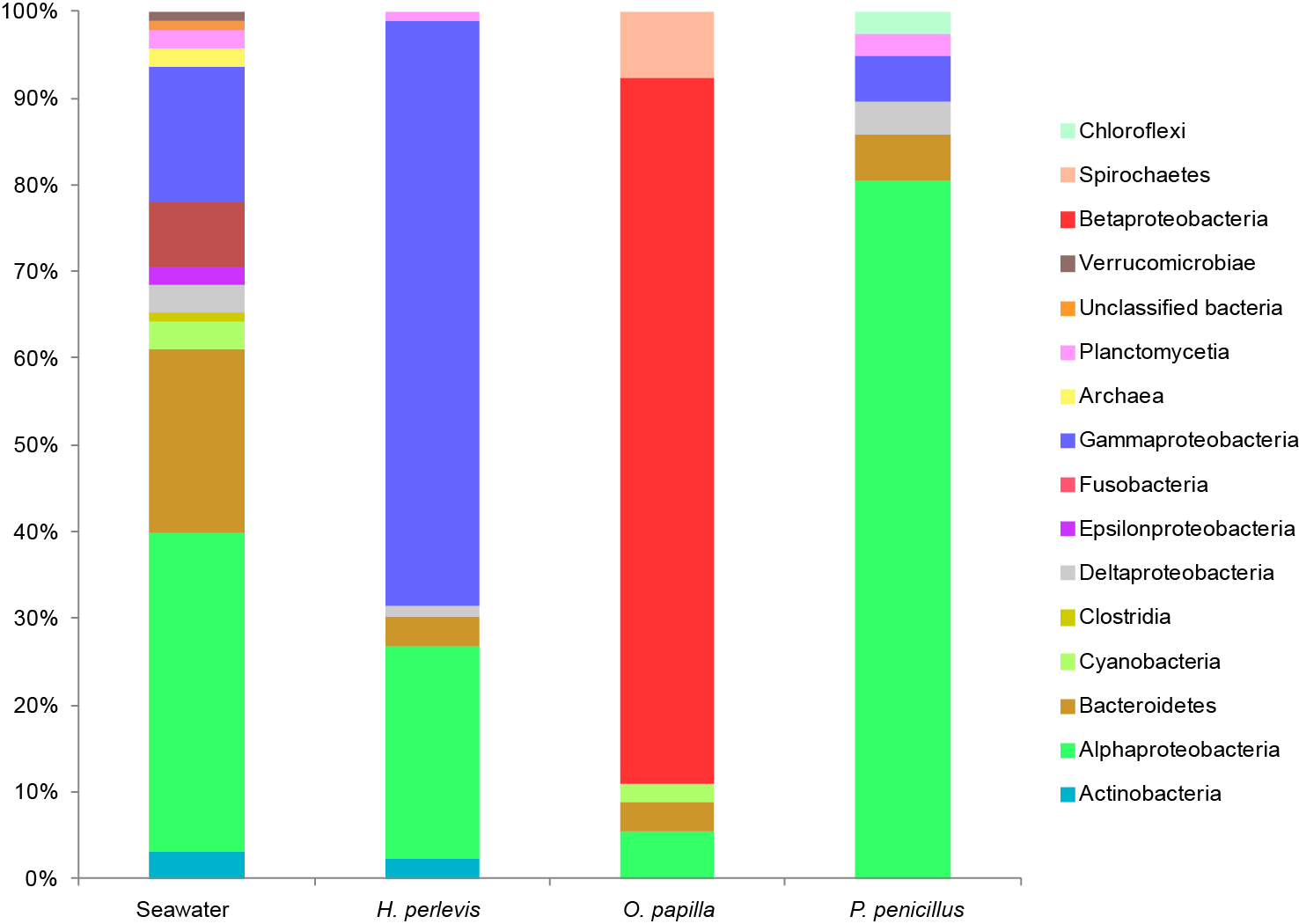


**Figure S2. Relative abundance of clones distributed in seawater and sponge hosts *H. perlevis*, *O*. *papilla* and *P*. *penicillus***. A total number of clones retrieved (n=349) from all source are classified at Domain/Phylum/Class level.
