# Supplementary figures and images for: Evidence of Unique and Generalist Microbes in Distantly Related Sympatric Intertidal Marine Sponges (Porifera: Demospongiae)
Source: PLoS One. 2013 Nov 12;8(11):e80653. doi: 10.1371/journal.pone.0080653 (PMC3827218; doi:10.1371/journal.pone.0080653)

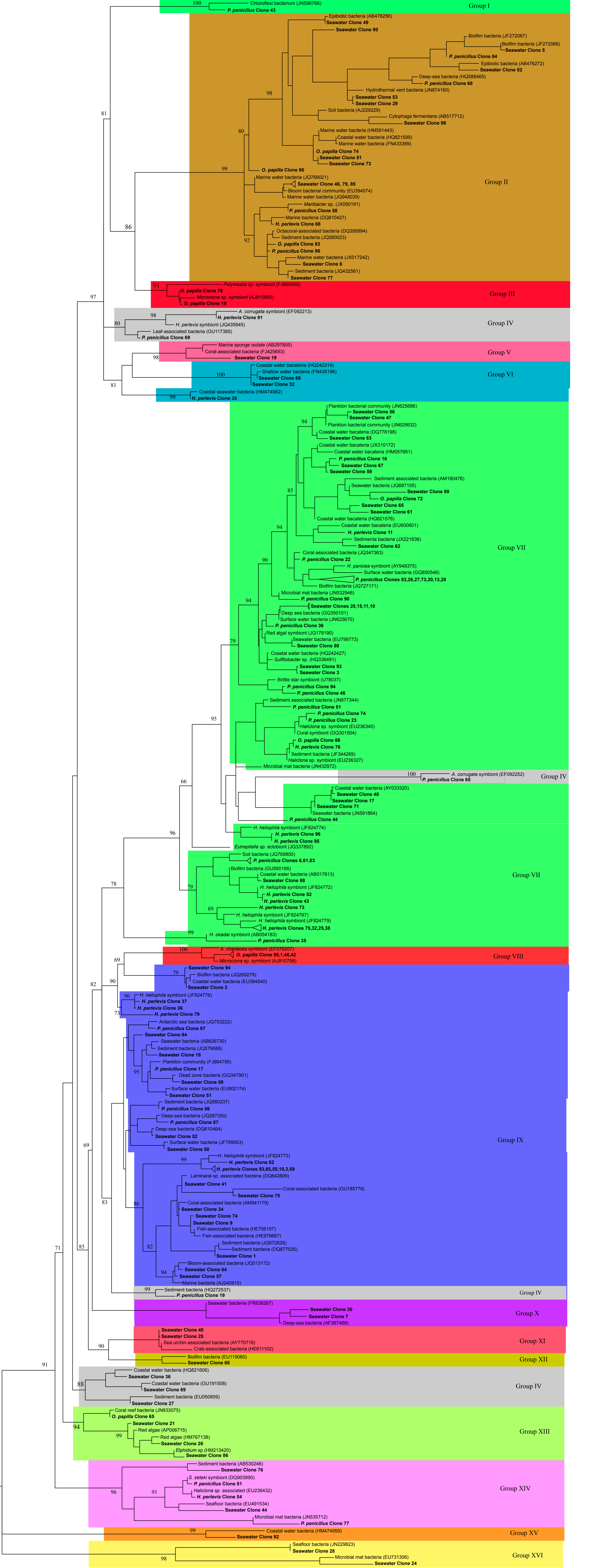

Supplement: Figure S3 — Maximum-likelihood phylogenetic tree constructed with partial 16S rRNA gene derived from sponge-associated bacteria. The closest relative retrieved from database was used for tree construction. Different bacterial groups are shown in colored rectangular box. Clone sequences derived from this study are represented in bold followed by the clone number. The clades are condensed (triangles) and bootstrap support values (%) are indicated. (PDF) [file pone.0080653.s003.pdf]
