# Supplementary material for: Evidence of Unique and Generalist Microbes in Distantly Related Sympatric Intertidal Marine Sponges (Porifera: Demospongiae)
Source: PLoS One. 2013 Nov 12;8(11):e80653. doi: 10.1371/journal.pone.0080653 (PMC3827218; doi:10.1371/journal.pone.0080653)
Supplement: Table S1 — Identified bacterial isolates from three sponges, H. perlevis , O. papilla and P. penicillus . The closest relative for each isolate was retrieved showing highest percent of similarity through BLAST search. (DOCX) [file pone.0080653.s004.docx]

| Host | Isolates | Order | Closest relative | % similarity |
| --- | --- | --- | --- | --- |
| *H. perlevis* | Isolate 1 | *Oceanospirillales* | *Cobetia marina* strain HNS037 | 100 |
|  | Isolate 2 | *Alteromonadales* | *Pseudoalteromonas* sp. RHS-str.402 | 100 |
|  | Isolate 3 | *Alteromonadales* | *Pseudoalteromonas* sp. RHS-str.402 | 100 |
|  | Isolate 4 | *Alteromonadales* | *Pseudoalteromonas* sp. RHS-str.402 | 100 |
|  | Isolate 5 | *Vibrionales* | *Vibrio* sp. H455 | 100 |
|  | Isolate 7 | *Vibrionales* | *Vibrio alginolyticus* strain J608 | 99 |
|  | Isolate 8 | *Vibrionales* | *Vibrio alginolyticus* strain J608 | 99 |
|  | Isolate 9 | *Vibrionales* | *Vibrio alginolyticus* strain J608 | 99 |
|  | Isolate 10 | *Vibrionales* | *Vibrio gigantis* strain S-50 | 100 |
| *O. papilla* | Isolate 1 | *Vibrionales* | *Vibrio* sp. H455 | 100 |
|  | Isolate 2 | *Vibrionaless* | *Vibrio gigantis* strain S-50 | 99 |
|  | Isolate 3 | *Vibrionales* | *Vibrio breoganii* strain C 4.15 | 100 |
|  | Isolate 4 | *Rhodobacterales* | *Sulfitobacter* sp. IS1 | 99 |
|  | Isolate 5 | *Vibrionales* | *Vibrio gigantis* strain S-50 | 100 |
|  | Isolate 6 | *Bacillales* | *Bacillus* sp. L07 | 100 |
|  | Isolate 7 | *Bacillales* | *Bacillus* sp. M71_S14 | 99 |
|  | Isolate 8 | *Vibrionales* | *Vibrio gigantis* strain S-50 | 99 |
|  | Isolate 9 | *Rhodobacterales* | *Pseudovibrio ascidiaceicola* strain NBRC 100514 | 99 |
|  | Isolate 10 | *Vibrionales* | *Vibrio gigantis* strain S-50 | 100 |
|  | Isolate 11 | *Vibrionales* | *Vibrio* sp. E505-4 | 99 |
| P. penicillus | Isolate 1 | *Vibrionales* | *Vibrio azureus* strain HNS036 | 99 |
|  | Isolate 2 | *Vibrionales* | *Vibrio alginolyticus* strain ZDS-6 | 100 |
|  | Isolate 3 | *Vibrionales* | *Vibrio alginolyticus* strain ZDS-6 | 99 |
|  | Isolate 4 | *Vibrionales* | *Vibrio gigantis* strain V007 | 99 |
|  | Isolate 5 | *Rhodobacterales* | *Pseudovibrio ascidiaceicola* strain NBRC 100514 | 100 |
|  | Isolate 6 | *Oceanospirillales* | *Hahellaceae* bacterium Ez249 | 95 |
|  | Isolate 7 | *Rhodobacterales* | *Roseobacter* sp. | 99 |
|  | Isolate 8 | *Bacillales* | *Bacillus* sp. LD121 | 99 |
|  | Isolate 9 | *Rhodobacterales* | *Pseudovibrio ascidiaceicola* strain NBRC 100514 | 100 |
|  | Isolate 10 | *Vibrionales* | *Vibrio* sp. K323 | 99 |
|  | Isolate 11 | *Vibrionales* | *Vibrio alginolyticus* strain ZDS-6 | 99 |

**Table S1. Identified bacterial isolates from three sponges, *H. perlevis*, *O. papilla* and *P. penicillus*.** The closest relative for each isolate was retrieved showing highest percent of similarity through BLAST search.
