# Supplementary material for: Evidence of Unique and Generalist Microbes in Distantly Related Sympatric Intertidal Marine Sponges (Porifera: Demospongiae)
Source: PLoS One. 2013 Nov 12;8(11):e80653. doi: 10.1371/journal.pone.0080653 (PMC3827218; doi:10.1371/journal.pone.0080653)
Supplement: Table S2 — Species richness, dominance, evenness and sampling coverage indices for bacterial communities from seawater, P . penicillus , H . perlevis and O . papilla (upper and lower confidence interval in parentheses). (DOCX) [file pone.0080653.s005.docx]

|  | Richness |  | Dominance | Evenness | Good’s coverage |  |  |  |  |
| --- | --- | --- | --- | --- | --- | --- | --- | --- | --- |
|  | *S_obs_* | *S_Chao1_* | *D_simpson_* | *E_1/D_* | *C* |  |  |  |  |
| Seawater | 72 | 300(174-580) | 0.005 (0.001-0.009) | 2.416 | 0.306818 |  |  |  |  |
| *P*. *penicillus* | 36 | 181(84-469) | 0.028 (0.007-0.049) | 0.969 | 0.423077 |  |  |  |  |
| *H. perlevis* | 26 | 45(32-88) | 0.039 (0.010-0.069) | 0.964 | 0.604651 |  |  |  |  |
| *O. papilla* | 12 | 15(12-34) | 0.195 (0.074-0.315) | 0.427 | 0.818282 |  |  |  |  |

**Table S2. Species richness, dominance, evenness and sampling coverage indices for bacterial communities from seawater, *P*. *penicillus*, *H*. *perlevis* and *O*. *papilla* (upper and lower confidence interval in parentheses).**
