# Supplementary material for: Evidence of Unique and Generalist Microbes in Distantly Related Sympatric Intertidal Marine Sponges (Porifera: Demospongiae)
Source: PLoS One. 2013 Nov 12;8(11):e80653. doi: 10.1371/journal.pone.0080653 (PMC3827218; doi:10.1371/journal.pone.0080653)
Supplement: Table S3 — Bacterial genetic diversity and community structure of sponges - O . papilla (OP), H . perlevis (HM), P . penicillus (PL) and seawater (SW). (DOCX) [file pone.0080653.s006.docx]

|  |  | SW-PL | SW-HM | SW-OP | PL-HM | PL-OP | HM-OP |
| --- | --- | --- | --- | --- | --- | --- | --- |
| LIBSHUFF | dC_xy_ | 0.042 | 0.057 | 0.062 | 0.043 | 0.062 | 0.107 |
|  | *P*-value_xy_ | *** | *** | *** | *** | *** | *** |
|  | dC_yx_ | 0.034 | 0.085 | 0.135 | 0.078 | 0.132 | 0.119 |
|  | *P*-value_yx_ | *** | *** | *** | *** | *** | *** |
| P-test | Score | 25 | 13 | 8 | 12 | 8 | 7 |
|  | *P*-value | ** | ** | ** | ** | ** | ** |
| AMOVA | Fs | 8.53 | 8.20 | 12.8 | 11.8 | 18.5 | 13.5 |
|  | *P*-value | ** | ** | ** | ** | ** | ** |
| HOMOVA | Bvalue | 0.73 | 1.84 | 1.16 | 0.27 | 0.11 | 0.01 |
|  | *P*-value | * | ** | ** | 0.123 | 0.244 | 0.573 |

**P* < 0.005, ***P* < 0.001, ****P* < 0.0001

Table S3 - Bacterial genetic diversity and community structure of sponges - *O*. *papilla* (OP), *H*. *perlevis* (HM), *P*. *penicillus* (PL) and seawater (SW).
